# Nicotine dependence as an independent risk factor for atherosclerosis in the National Lung Screening Trial

**DOI:** 10.1186/s12889-019-6419-8

**Published:** 2019-01-22

**Authors:** Junjia Zhu, Kevin Nelson, Jennifer Toth, Joshua E. Muscat

**Affiliations:** 1 Penn State College of Medicine, Department of Public Health Sciences, Pennsylvania State University, MC CH69, 500 University Drive, P.O. Box 850, Hershey, PA 17033 USA; 2 Penn State Milton S. Hershey Medical Center, Department of Pulmonary Medicine, 500 University Drive, PO Box 850, Hershey, PA 17033 USA

**Keywords:** Atherosclerosis, Tobacco smoking, Tobacco use disorder, Risk factors

## Abstract

**Background:**

Atherosclerosis and COPD are systemic inflammatory diseases that share common risk factors including cigarette smoking. A high level of nicotine dependence is emerging as a recently identified risk factor for pulmonary impairment, chronic obstructive pulmonary disease and tobacco-related cancers. We hypothesized that nicotine dependence is associated with the risk of atherosclerosis in long-term cigarette smokers.

**Methods:**

A nested case-control study was conducted within the National Lung Cancer Screening Trial- American College of Radiology Imaging Network. Cases were defined as having a new diagnosis of any type of atherosclerosis. Controls were matched on a 2:1 basis by age, sex, race, study center, smoking status, years of smoking, and frequency of smoking. Dependence was measured by the time to first cigarette after awakening (TTFC).

**Results:**

The study included 166 cases and 286 controls. Compared to participants who smoked within 5 min after waking, the risk of atherosclerosis for participants who smoked an hour or more after waking was borderline non-significant (odds ratio = 0.49, 95% confidence intervals [CI] 0.23, 1.00). Findings were similar for men and women. For aortic atherosclerosis, the corresponding odds ratio was 0.24 (95% CI 0.08, 0.69). Hypertension was associated with an increased risk and body mass index was associated with a decreased risk of aortic atherosclerosis. The TTFC was unrelated to coronary atherosclerosis.

**Conclusions:**

Compared to smoking immediately after waking, delaying an hour or more reduces the risk of aortic atherosclerosis even among long-term heavy smokers. Possible mechanisms that explain this association are intensity of smoking, inflammation and oxidative stress, and elevated lipid levels.

## Background

Cigarette smoking is an established cause of the clinical consequences of atherosclerosis including peripheral arterial disease and coronary artery disease [1]. In a meta-analysis of 55 studies, the relative risk of peripheral arterial disease (PAD) associated with cigarette smoking was 2.71 (95% CI 2.28 to 3.21, *p* < 0.001) [2]. The risk for coronary heart disease is similar [3].

Cigarette smoking also causes atherosclerosis. In the Multi-Ethnic Study of Atherosclerosis, cigarette smoking increases the risk of aortic and coronary atherosclerosis, with higher risks associated with aortic atherosclerosis [4]. Smoking also increases the pathogenic potential of atherosclerosis by increasing the thickness of intimal-medial thickness of the arteries [5, 6]. The association is dose-dependent with pack-years of exposure [5]. A history of smoking and especially heavy smoking is associated with the presence and size of aortic atherosclerotic lesions [7–10] although the findings for coronary atherosclerosis are not entirely consistent [11].

Recently, a literature has been emerging that nicotine dependence itself, independent of the amount of smoking, is a strong factor for several major causes of mortality from tobacco-related diseases. Using the measure time to first cigarette after waking, which is often considered the best single indicator of nicotine dependence [12], a later time to first cigarette (TTFC) was found to decrease the risk of smoking-associated outcomes including COPD [13], pulmonary impairment [14], lung cancer [15–17] and other cancers [18]. When smokers delay having their first morning cigarette, their risks are reduced compared to smokers who take their first cigarette within minutes after waking, even after adjustment for smoking history. The reasons for these associations are not well understood but they have been consistently shown for both malignant and nonmalignant conditions.

Consequently, we were interested in determining whether nicotine dependence itself is a possible independent predictor of atherosclerosis. This may provide insights into the etiology of heart disease, but also has potentially important public health implications in identifying smokers who are at the greatest risk and targeting prevention programs towards them.

## Methods

The National Lung Screening Trial (NLST) was conducted to compare the detection rates of lung cancer using low-dose helical (spiral) computed tomography (CT) vs. standard chest X-ray in a high-risk population of over 53,454 middle aged to elderly current or former heavy smokers. The methods and materials including consent procedures have been previously described in detail [19], including questionnaire data on age, sex demographics and self-reported medical conditions such as hypertension and diabetes. The NLST population was self-referred. As part of the NLST, a separate protocol was conducted by the American College of Radiology Imaging Network (ACRIN) to develop and maintain a biomarker repository to test biomarkers that assist or replace imaging for early detection of lung cancer. Serial blood, sputum, and urine samples were collected from 10,300 NLST ACRIN participants. The ACRIN participants also completed an additional questionnaire that included an assessment of nicotine dependence, including questions on smoking after waking. (A PDF version of the questionnaire is available via ACRIN website at https://www.acrin.org/Default.aspx?tabid=282). High resolution computed tomography has been used for screening coronary artery calcium, but chest CT performed for lung cancer screening has been adapted for coronary calcium detection [20] and can reveal a number of other abnormalities for conditions other than lung cancer. Radiographically detected coronary artery calcification in the NLST and CT screening in general using chest CT can detect the presence and extent of subclinical atherosclerotic plaques which potentially could be used to develop medical intervention strategies to prevent the occurrence of future cardiovascular disease [21, 22]. Chest CT can image the entire heart whereas CT methods performed specifically for coronary calcium have a more limited visual view. However chest CT is not conducted for individuals who do not meet the screening guidelines of chest CT [23]. In our data, 220 of 14,028 (about 1.57%) NLST –ACRIN participants were diagnosed with any type of atherosclerosis.

A matched case-control study was created from NLST files made available for third party use, which represent 90% of total NLST subjects. Fifty-five participants were diagnosed with ICD-9 code 440 [aortic atherosclerosis], and 40 cases were coded as having both 440 and ICD-9 code 414 [coronary atherosclerosis]). Seventy-one participants were coded as having 414 only. Although there is limited data, the pathologic process and effect of risk factors for coronary atherosclerosis may differ from that for atherosclerosis in the abdominal location of the aortic artery, where most aortic aneurisms occur [24]. Consequently, the analysis also included aortic and coronary atherosclerosis in subgroup analysis. Control subjects were randomly selected by a 2:1 ratio to cases from participants without atherosclerosis. Controls were matched to cases by age (+/− 5 years), sex, race, study center, smoking status (current/ex-smoker), years of smoking (+/− 2 years) and cigarettes per day (+/− 2). A total of 166 cases and 286 matched controls were included in the dataset. The matching process was performed using R program language version 3.4.4 (R Foundation). Base R software via direct programming was used for matching and no specific matching packages were used.

### Statistical analysis

Conditional logistic regression was conducted to determine the relationship with TTFC using SAS statistical software version 9.4 (SAS Institute, Cary, NC, USA). Indicator variables were used to code the associations using TTFC≤5 min as the reference category, relative to the categories of 6–29 min, 30–60, and > 1 h. This reference category was selected since the there were relatively few subjects who smoked > 1 h and the goal was to determine if delayed smoking is a potential preventative behavior. An alternative model substituted the Fagerstrom Score, which can be calculated from the 6-item Fagerstrom Test for Nicotine Dependence [25] and includes TTFC as one of its most heavily weighted items.

Initial models examined the potentially confounding effects of other tobacco factors including cigarette brand (e.g. Menthol (yes/no), pipe use (ever/never), and cigar use (ever/never). None of these factors were predictive of atherosclerosis risk. The final models were adjusted for body mass index (continuous), reported past diagnoses of diabetes (categorical), hypertension (categorical) and myocardial infarction (categorical). Matched odds ratios (OR) and 95% confidence intervals (CI) were determined. Separate models were developed for ICD-9414, and for ICD-9440 (includes participants with co-diagnosis of 414). All statistical tests were two-sided and the significance level used was 0.05.

## Results

The basic demographic characteristics of the participants are shown in Table 1. Participants were very similar with respect to the levels of the matching variables. About 40% of participants were women and 99% were white. Over 36% held college degrees.

**Table 1:** Baseline characteristics of cases and matched controls, NLST-ACRIN participants

	Cases *N* = 166	Controls *N* = 286
Mean age	63.0	62.6
Sex (female, %)	39.8	38.5
Race (white, %)	98.8	98.6
Years of Education (%)		
< 8	1.8	1.1
9–11	4.8	5.4
12	21.8	23.7
13–16	31.5	33.4
≥ 16	40.0	36.3
Smoking status (current %)	51.8	53.0
Mean cigarettes per day	27.1	26.7
Mean pack-years of smoking	56.7	55.4
Menthol (%)	16.3	21.3
TTFC (%)		
≤ 5 min	38.0	31.1
6–29 min	36.1	41.0
30–60 min	15.1	12.4
> 60 min	10.8	15.6

Table 2 shows the relationship between TTFC and atherosclerosis. Compared to subjects who smoked ≤5 min after waking, there was no significant relationship between categories of TTFC and atherosclerosis risk. The risk for the greatest contrast, i.e. > 60 min, was 0.49 (95% CI 0.24, 1.00). In an alternative model replacing TTFC with the Fagerstrom Score, the score variable was not significant (*p* = 0.32). A significant association was observed for hypertension (OR = 1.78) but not for BMI, diabetes and myocardial infarction. In sex-specific analyses, the results were similar for men and women.

**Table 2:** Time to first cigarette and other risk factors for atherosclerosis in long-term heavy smokers

Effect	Odds ratios and their 95% CIs		
	All matched samples N = 166 cases, 286 controls	Aortic Atherosclerosis^a^ *N* = 95 cases, 164 controls	Coronary atherosclerosis only *N* = 71 cases, 122 controls
TTFC			
≤ 5 min	Reference	Reference	Reference
6–29 min	0.72 (0.44–1.16)	0.54 (0.27–1.10)	0.9 (0.45–1.82)
30–60 min	0.94 (0.49–1.82)	1.08 (0.46–2.52)	0.9 (0.29–2.74)
> 60 min	0.49 (0.24–1.00)	0.24 (0.08–0.69)	1.27 (0.43–3.74)
BMI	0.95 (0.91–1.00)	0.93 (0.86–0.99)	0.98 (0.91–1.05)
Diabetes	1.45 (0.79–2.64)	0.58 (0.20–1.68)	2.58 (1.16–5.76)
Hypertension	1.78 (1.15–2.74)	2.34 (1.25–4.38)	1.36 (0.72–2.59)
Heart Attack	1.22 (0.70–2.13)	1.58 (0.71–3.52)	0.98 (0.42–2.29)

All estimates were adjusted for the other covariates in the model. ^a^ Includes 40 cases with co-diagnosis of coronary atherosclerosis

The risks were examined separately for subjects with aortic atherosclerosis (ICD 440 and 440 + 414). Compared to subjects who smoked ≤5 min after waking, there was no significant relationship with the TTFC categories 6–29 min and 30–60 min. The risk of having aortic atherosclerosis for those with TTFC > 60 min was significantly lower with OR = 0.24 (95% CI 0.08, 0.69). There was no association between diabetes and heart attack with aortic atherosclerosis. The risk associated with hypertension (OR = 2.34) was statistically significant. There was a significant inverse association between BMI and risk (OR = 0.93). Limiting the analysis to ICD 440 only, the associations were very similar. For example, the odds ratio for TTFC > 60 min was 0.30 (95% CI 0.08, 1.06). None of the TTFC categories were associated with coronary atherosclerosis (Table 2). None of the other medical risk factors were associated with coronary atherosclerosis, except for diabetes (OR = 2.58).

## Discussion

These findings show that a behavioral measure of nicotine dependence, the TTFC, is significantly associated with the risk of aortic atherosclerosis among long-term heavy smokers. Subjects who delayed smoking for more than 60 min had a 0.24 risk compared to subjects who smoked within 5 min after waking. Atherosclerosis is an asymptomatic progressive disease that may become symptomatic with the development of pain in the chest or extremities, or loss of physical function. The cases that were newly diagnosed in the NLST are assumed to be asymptomatic. An analysis of ICD codes from the medical evaluation of subjects in this dataset did not find symptomatic conditions associated with advanced atherosclerosis (e.g. angina).

There is evidence that the level of nicotine dependence in smokers, as measured by TTFC and the Fagerstrom Test for Nicotine Dependence, is associated with cardiovascular disease and respiratory disorders in the National Health and Wellness Survey [26]. The findings are cross-sectional and represent comorbid conditions rather than causal associations. The specific cardiovascular diseases were not described but the findings suggest that like COPD and cancer, less addicted smokers have lower rates of cardiovascular and respiratory disease. The current findings from NLST-ACRIN appear to be consistent in that a later time to first cigarette is associated with a reduced risk of aortic atherosclerosis, which is an independent predictor of cardiovascular disease events [27]. The association in this study is apparent even after careful matching for cigarette smoking history. The association between TTFC and atherosclerosis was found specifically for aortic atherosclerosis and not for coronary atherosclerosis. This is consistent with other data that examined the risks of smoking history and atherosclerosis. In the Multi-Ethnic Study of Atherosclerosis, the strength of risk factors differed between aortic and coronary atherosclerosis, with higher risks associated with aortic atherosclerosis [4]. For smoking, current smokers in that study had a 3.3 increased risk of aortic atherosclerosis compared to never smokers, and a 1.51 increased risk for coronary atherosclerosis.

The strong statistical associations between early TTFC and lung and other cancers have more recently been extended to COPD and, at least in this study, aortic atherosclerosis. The mechanisms underlying these associations are unknown, but are noteworthy in that a simple measure of behavior has a strong association with a number of distinct pathologies. The TTFC appears to be a predictor of both malignant and nonmalignant smoking-associated disease. One possible explanation for these findings is that the TTFC may reflect the intensity and dose of smoking, and is strongly correlated with biomarkers of tobacco smoke constituents [28, 29]. The mechanisms of cigarette smoke-induced cardiovascular disease are not well understood but likely involve inflammation [30]. Smoking cessation and lighter vs. heavier smoking is associated with lower markers of inflammation such as glutathione and myeloperoxidase [31, 32]. Nicotine dependence itself, independent of smoking amount, may have pro-inflammatory effects. Higher levels of nicotine dependence are found in persons with psychiatric comorbidities including major depressive and mood disorders [33]. Depression is associated with an increase in oxidative stress and damage to neurological processes. Depressed smokers have significantly higher levels of oxidative and inflammatory biomarkers than non-depressed smokers [34]. Under this hypothesis, an alternative explanation for smoking intensity as the cause for an earlier TTFC increasing (aortic) atherosclerosis risk would be increased oxidative stress associated with stress or depression that characterizes higher levels of nicotine dependence. An early time to first cigarette has also been related to lipid levels. In an analysis of four consecutive waves of the National Health and Nutrition Survey (NHANES), an earlier TTFC was significantly associated with lower HDL levels, a lower LDL/HDL ratio [35]. An earlier TTFC was also associated with having high cholesterol levels (> 200 mg/dL). The mechanism for this association is unknown. In experimental animals, oral nicotine increases LDL and decreases the HDL/total cholesterol ratio through impaired clearance of LDL from the plasma [36, 37]. A forth possible explanation as an alternative to TTFC as a marker of smoking intensity is that a cigarette smoked immediately after waking is more toxic than when smoked later in the day, raising the intriguing hypothesis that delaying the first cigarette smoked for an hour or more may substantially decrease the risk of aortic atherosclerosis. Our data indicate a significant decrease in risk when delaying for an hour or more. Delaying for less than one hour had no beneficial effect. Delaying the first cigarette could be a potential preventative strategy. Cigarette smoke exposure leads to an adaptive response characterized by increased synthesis of the major intracellular antioxidant, glutathione (GSH). Evidence suggests diurnal variation in GSH production in mammals [38]. Human studies are needed to determine if GSH response to smoking varies by time after waking.

In other findings from this study, the association of a decreased risk of aortic atherosclerosis with increasing body mass index confirm a recent autopsy series that higher BMI was associated with less severe aortic atherosclerosis [39]. BMI was unrelated to coronary atherosclerosis in the current study. There are mixed findings from forensic data on obesity and lower incidence/severity of coronary atherosclerosis [40, 41].

Strengths of the study included the very large data set that allowed for multiple and highly refined matching criteria that allowed us to isolate the independent effect of TTFC. Controls were matched to cases on not just a range of smoking history variables but to a very tight range for number of cigarettes per day. This process minimizes any residual confounding due to smoking history. We further controlled for major risk factors for atherosclerosis including body weight, diabetes, hypertension and heart attack. However information on exercise and diet was not collected and it was not possible to control for these potential confounders. Information was collected on alcohol use but was missing for many of the participants. The data on atherosclerosis medical risk factors such as hypertension and diabetes was based on self-report and not a medical diagnosis. However validation studies in United States populations have shown reasonable agreement between self-reports and medical diagnosis for hypertension, MI, and stroke (κ = 0.71–0.80) [42], although agreement for hypertension has also been reported to be fair (k = 0.25) in other surveys [43]. Numerous studies have found a very high degree of accuracy of self-reported diabetes [44–46]. Atherosclerosis itself may be misdiagnosed and the ICD code for atherosclerosis, might represent a different underlying pathology. Although rare, scleroderma and Buerger’s Disease cause hardening of the arteries.

Another possible limitation of the current study is that the NLST participants were highly educated. About 40% were college graduates, whereas smoking rates in the U.S. are very low in adults with a college degree. In addition, since the ACRIN cohort sub study was conducted in a subset of the study centers, the ACRIN participants may not be representative of all NLSS participants. Previous analysis of the ACRIN cohort subset vs. the entire NLSS showed no meaningful differences by age, sex, smoking history and other demographic variables [47]. Almost all participants were white, limiting our ability to generalize the findings to other racial and ethnic groups. In addition, lung cancer screening guidelines are limited to smokers ages 55–79. We cannot make inferences about the relationship between TTFC and atherosclerosis in younger and older ages.

## Conclusions

Atherosclerosis is a progressive disease that is asymptomatic for decades. It is commonly diagnosed by angiography following severe symptomology, where advanced stenosis occurs later in life. Coronary artery calcification is insignificant in the early stages of atherosclerotic lesions. It is more advanced with late stage atherosclerosis and in older age. For the purposes of the current study, the results for TTFC are potentially more valid than if study subjects were identified from a diagnostic procedure where patients would include only those with more advanced lesions and were symptomatic. The current study indicates that even among lifetime heavy smokers, the risk of aortic atherosclerosis varies by time to first cigarette. Whether delaying the TTFC could reduce the risk would need to be determined in a clinical trial setting.
